# A biomechanical study of spherical grip

**DOI:** 10.1186/2193-1801-2-591

**Published:** 2013-11-04

**Authors:** Jaime Martin-Martin, Antonio I Cuesta-Vargas

**Affiliations:** Faculty of Health Science, Department of Physiotherapy, University of Malaga, Malaga, Spain; School of Clinical Science, Faculty of Health Science, Queensland University of Technology, Brisbane, Australia

**Keywords:** Electromyography, Accelerometer, Spherical, Grip, Signal processing

## Abstract

Use of the hand is vital in working life due to the grabbing and pinching it performs. Spherical grip is the most commonly used, due to similarity to the gripping of a computer mouse. Knowledge of its execution and the involved elements is essential. Analysis of this exertion with surface electromyography devices (to register muscular activity) and accelerometer devices (to register movement values ) can provide multiple variables. Six subjects performed ball gripping and registered real-time electromyography (thenar region, hypothenar region, first dorsal interosseous, flexors of the wrist, flexor carpi ulnaris and extensors of the wrist muscles) and accelerometer (thumb, index, middle, ring, pinky and palm) values. The obtained data was resampled “R software” and processed “Matlab Script” based on an automatic numerical sequence recognition program. Electromyography values were normalized on the basis of maximum voluntary contraction, whilst modular values were calculated for the acceleration vector. After processing and analysing the obtained data and signal, it was possible to identify five stages of movement in accordance with the module vector from the palm. The statistical analysis of the variables was descriptive: average and standard deviations. The outcome variables focus on the variations of the modules of the vector (between the maximum and minimum values of each module and phase) and the maximum values of the standardized electromyography of each muscle. Analysis of movement through accelerometer and electromyography variables can give us an insight into the operation of spherical grip. The protocol and treatment data can be used as a system to complement existing assessments in the hand.

## Background

Gripping and pinching are basic functional exertions of the hand, and are used continuously in the activities of daily life and work (Murgia et al. 2004). Office work involves different tasks based on functional gripping. An example of this is the three-tip tripod grasp to hold a pen in writing (Gentilucci et al. 2003) and the spherical grip to move a computer mouse (Visser et al. 2004), in both cases adapted to the shape of the objects.

The movements made by the hand and arm during mouse grip have been analysed from different perspectives. The involvement of the extensors of the index and middle fingers in relation to the design of the mouse has been analysed on the basis of electromiographical findings (Lee David et al. 2007). The study also focused on the area of contact of the hand and wrist with the mouse (Kang et al. 2012), as well as other elements of the arm in relation to tasks performed with the mouse, and how the elements involved behave in this task (Chen et al. 2012; Laursen and Jensen 2000). Sphercial grip is also used to open bottles or grap a tennis ball among ther tasks.

The application of real-time reading equipment such as electromyography (Merletti et al. 2010), registration of muscle activity, inertial sensors (Cuesta-Vargas et al. 2010) and movements made in the tasks can provide more information. The use of such equipment with the hand is possible thanks to the body adapting to the segment where it is placed. In surface electromyography (sEMG) such adaptation depends on the placement of the electrodes (Mesin et al. 2009). For the inertial sensors, there are gloves with these devices built-in in order to directly transmit the information registered (AcceleGlove 2011; MediTouch - HandTutor™ 2012; Overview | cyberglovesystems.com 2012).

The use of these devices has focused on the recognition of gestures and sign language (Li et al. 2011; Wenhui et al. 2009), registration of functional activity (Bonato et al. 2004; Roy et al. 2010) and the classification of the hand movement (Fougner et al. 2011), among others.

To our knowledge, there are no studies of muscle activity of the surface (sEMG) together with the accelerations carried out by the hand during mouse gripping or, failing this, spherical gripping.

Therefore, the aims of this study are to: parameterize the spherical grip in relation to the instruments of accelerometer and electromyography; process and analyse the different variables of the movement; identifiy those variables which provide greater relevance as complementary factors to the evaluation of the movement. This analysis will provide a more objective view of the most suitable position for hand grip on the ball or spherical grip.

## Material and method

### Study objective

Quantitative, non-experimental, analytic, transversal approach, aimed at detecting functionality variables of spherical grip, forming a new research pilot study.

### Subjects

A sample of 6 healthy adults was selected for this study. The inclusion criteria were: between the ages of 18 and 35 years, no previous pathologies, not suffering of motor disturbances in the upper right extremity, no effects on the skin, right hand dominant, accept and sign the informed consent. Exclusion criteria were established: dominance of the left upper limb, affectations of the locomotor system, and any other which does not meet the inclusion criteria.

### Ethical approval

Ethical approval was given by Committee of Research of the Faculty of Health Science at Malaga University.

### Material

The data collection instruments for the variables were classified in four groups: a) anthropometric variables: height and weight b) dynamometry; c) clinimetric variables: the Upper Limb Functional Index (ULFI) (Gabel et al. 2006) and the QuickDASH (Hervás et al. 2006); d) monitored variables: d.1) accelerometer using the Acceleglove device (AnthroTronix, Inc) (AcceleGlove 2011) with Acceleglove Visualizer registering software d.2) the sEMG recorder MEGA ME 6000 (Mega Electronics Ltd | Pioneers in Biosignal Monitoring Technology 2011), in conjunction with specialised software for capturing and processing data provided by the manufacturer MegaWin 3.0.1.

Anthropometric variables were obtained in accordance with the following parameters: height measured in metres rounded to two decimal points, and weight measured in kilograms using the described procedure on ISAK(Stewart et al. 2011).

The dynamometer used was the Jamar Hydraulic Hand Dynamometer manufactured by Sammons Preston Rolyan (Patterson Medical - Evaluation 2012), activated with palm pressure. The force of palm pressure was measured in kilograms/cm^2^. The Jamar Hand Dynomometer was adjusted to fit the metacarpal readings.

The clinimetric variables used were those of the Upper Limb Functional Index (ULFI) (Gabel et al. 2006; A. ICuesta-Vargas Antonio and Gabel 2013) and the QuickDASH (Hervás et al. 2006). The correlation index of the ULFi to the DASH was r = 0.85; Confidence Interval, CI, 95%, demonstrating test-retest reliability with an intraclass correlation coefficient of 0.96 and 95% CI, consisting of twenty-five items in the Spanish version (Gabel et al. 2006). The internal consistency of QuickDash (The QuickDASH | DASH 2012) registered a Cronbach Alpha of 0.94; the test-retest reliability of 0.94 comprised three parts: general (11 items), work (4 items) and sport or music (4 items). In both questionnaires a higher score was indicative of a major grade of disability.

**Figure 1:**
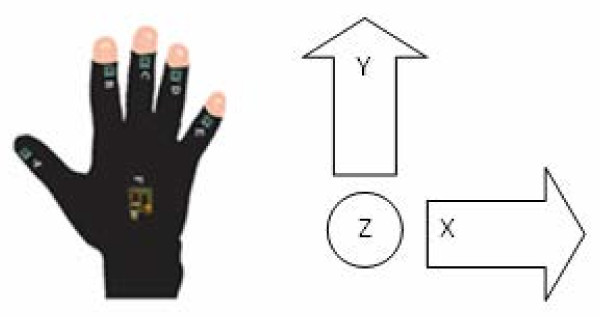
**Acceleglove output signal convention (top view).**

The accelerometer type variables were registered using the Acceleglove device (ACC) (AnthroTronix, Inc) (AcceleGlove 2011). The AcceleGlove is a lycra glove fitted with six inertial accelerometer sensors, one on the back of each finger on the middle phalanx and a sixth sensor on the back of the palm. The software used for registering and capturing data was the Acceleglove Visualizer supplied by the manufacturer. The sampling rate of the device was 120 Hz. Each accelerometer has three axis positions (X, Y, Z) with a precision range of ±1.5 g. The reading is provided on “g”, this being the unit of standard gravity or standard acceleration due to free fall, nominal gravitation acceleration. The axis correlation of the glove is illustrated in Figure 1. If the hand is in horizontal position, the Z axis is the gravity vector which is perpendicular to the surface of the earth. The X and Y axes are perpendicular to each other and to Z. The hardware provides the following acceleration variables measured in“g”: thumb, index, middle, ring, pinky and palm in three spatial directions (X, Y, Z) together with the time reading in Unix 1 January 1970 (LLC, Books 2010). On the basis of these parameters the following data were obtained as indirect variables: time in seconds and module vector. On the basis of the variables obtained by this device, the fragmentation of movement was calculated both at maximal and minimal levels, not only with regards to the exertion performed but also at each phase of the movement. The variation between minimum and maximum was calculated.

- Time: obtained in seconds based on the Unix reading registered by the device 1 January 1970 LLC, Books (2010). The calculation was made by subtracting the accumulated figure from that established in the first reading registered by the device.
- Module Vector Acceleration: obtained from the root of the squared values on the axes sum: . The operation was performed on the “x”, “y” and “z” axes of each of the accelerometers corresponding to the thumb, index, middle, ring, pinky and palm. It is therefore the longitude of the vector or dimension of the acceleration.

SEMG variables were registered using the MEGA ME6000 (Mega Electronics Ltd | Pioneers in Biosignal Monitoring Technology 2011), MT-M6T8-0-10 (Kuopio), in conjunction with the specialist software for capturing and processing data provided by the manufacturer MegaWin 3.0.1, measured in microvolts (μV); the sampling range of the device was 1000 Hz. The muscle region measured and registered for the spherical grip was: hypothenar muscles (opponens digiti minimi), thenar muscles (flexor pollicis brevis), first dorsal interosseous muscle (first dorsal interosseous), flexors of the wrist (palmaris longus), flexor carpi ulnaris muscle and the extensors of the wrist (extensor carpi radialis). Tests of maximum voluntary muscle contraction(MVC) (Jacqueline Montgomery 2007) were performedin order to determine the maximum value of the electromyography muscle region. Data capture was obtained using Electrocardiogram Lessa electrodes (Registration Papers Lessa - Products 2012) in two sizes: child size for the hand and adult size for the forearm area. Child electrodes were used due to their smaller adhesive, which makes it easier to position them on the hand The placing and positioning of the electrodes was carried out in accordance with the bibliography (Perotto and Delagi 2005), (Hermens et al. 1999) and the accompanying software for the device Megawin 3.0.1 was used to register the obtained data. A protocol for recording in RAW image format was adopted consisting of the aforementioned muscles for each subject. The acquisition and processing of the signal was carried out using the same software. The sEMG variables were: maximal, minimal and normalisation of each muscle.

A specific device was used in order to ensure synchronisation of the equipment and fulfil the parameterising condition in real time and register data simultaneously: Digital Video Trigger manufactured by Mega Electronics Ltd. (Kuopio, Finland). A trigger, when pressed, placed a marker on the sEMG to indicate the start of the recording for the AcceleGlove Visualizer; when pressed a second time, a second marker indicated the end of the recording for the AcceleGlove software. This device ensured correct interaction between the different equipment in compliance with the parametrisation condition in real time for both items of equipment, which started and finished registering data simultaneously.

### Method

**Figure 2:**
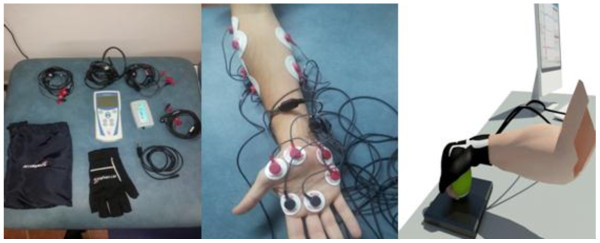
**Experimental set-up equipment (left), electromyography (centre), grip (right).**

The participants performed the functional exertion using spherical grip (the action consisted of gripping a tennis ball which is fixed to a piece of wood). The exertion was repeated three consecutive times and the data registered. The subjects performed the test while seated on a chair 50 cm high, with a straight back and the arm held close to the body and the elbow bent at 90°. The assessment table was placed opposite the subject on a flat surface 75 cm high. A reference mark was placed on the assessment table on which the middle finger of the right hand was placed prior to the start of the test. Each of the subjects remained in the aforementioned position for a period of 4 seconds, after which an acoustic alert prompted them to move their hand to the area indicated in order to carry out the exertion using spherical grip. Four seconds after the first signal, a second alert prompted them to return to the starting position. The procedure was performed in a series of three repetitions, see Figure 2 of the experimental set-up.

### Theory

The spherical grip was parameterised based on the aforementioned protocol for the exertion using the right hand. In accordance with the data registered by the Acceleglove Visualizer software, 18 direct variables were obtained from one single reading, based on the obtained data from each of the six accelerometers with triple-axis direction (X, Y, Z). On the basis of the results obtained, two new indirect variables were established: “time” and “module vector”.

While performing spherical grip, five sub-phases were identified due to significant variations produced by the acceleration vectors. These variations have a direct correlation with the various sub-phases of the exertion (T1-T5), corresponding to the movements and positions adopted by the hand. In order to fragment the task, a quantitative criterion was established according to the sub-phase of the hand movement (static or dynamic). To determine the rest phases, the script parses the basis of numerical data in search of the three values which are most repeated in three different sections, and then generates a range of stability and a repetition environment for these values. The stability range was determined around the value which is repeated most times in each static section. This range was defined by the production of ten consecutive registers with approximate values around the most repeated over a range of ± 2 units based on a smooth original signal.

These phases were determined on the basis of the module vector of the palm. In order to identify two type of phases, static phase and dynamic phase, quantitative criteria were established based on Matlab Script (MathWorks España - MATLAB 2013). In the static phase the module vector of palm acceleration remained constant, unlike in the dynamic phase.

In accordance with the quantitative criteria established, five phases of spherical grip were identified. The sequencing of these phases was as follows: T1 or repose (static), the hand remains static awaiting the acoustic signal; T2 or calibration (dynamic), the hand moves to the area indicated to perform the spherical grip after the acoustic signal is heard; T3 or success (static), the hand performs the spherical grip in the area indicated; T4 or return (dynamic), the hand moves to the initial position after the acoustic signal is heard; and T5 or repose (static). A fragmentation of the movement by exertions and temporal sub-phases was obtained on the basis of these results.

The six sEMG data muscle were processed using MegaWin software based on data obtained in RAW image format, subjected to a filter defined as transient bandwidth 20.00 Hz, attenuation 60.00 dB and high frequency 400 Hz. An RMS was applied to the results obtained after filtering (mean adjusted frame width = 0.01 s).

The data registered by both devices (Acceleglove and Mega ME6000) was combined in a database using the R software (The R Project for Statistical Computing 2012), adjusting mean values according to the unit of time measured in each case in order to integrate both readings correctly.

**Figure 3:**
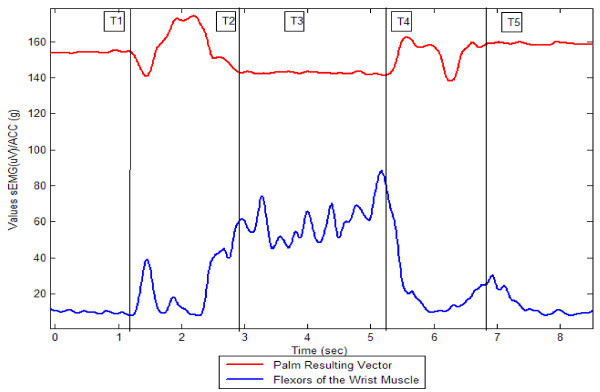
**Spherical grip of one subject by phase.**

**Figure 4:**
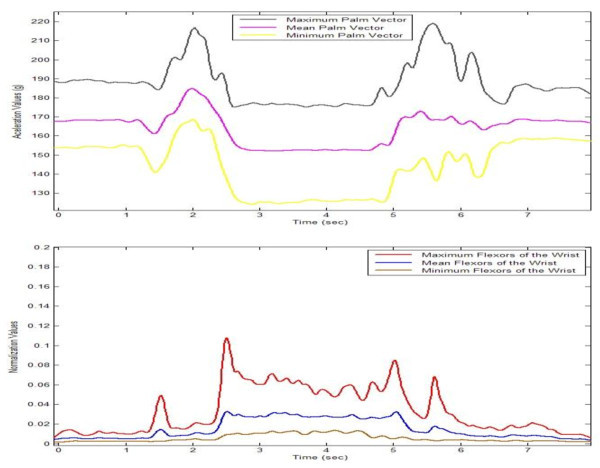
**Range Values of Palm Module Vector (up) and Flexors of the Wrist Muscle Normalized (down).**

Figure 3 represents the temporal spectrum of a subject while performing the spherical grip based on sEMG variables of the flexors of the wrist and the module palm vector of the ACC values throughout the sequence, based on data produced by Matlab Software. The data for both variables was set to smoothing spline at 0.9997 specification, in order to obtain a more uniform curve. Figure 4 uses the same procedure.

### Statistical analysis

A descriptive statistical analysis was carried out based on the means and standard deviations of the variables with the highest relevance for the analysis of the spherical grip. The variables of most interest to the analysis were obtained indirectly from the sEMG (normalised values) and the accelerometer (variation of the acceleration). Work posture analysis was carried out by independent movement phases.

## Results

Table 1 shows the descriptive statistics of the selected sample, on the basis of the anthropometric variables, dynamometric and clinimetric values, mean values and standard deviation.

**Table 1:** **Descriptive analysis of the sample**

	N	Mean ± SD
Age	6	25.50 ± 4.03
Height (m)	6	1.70 ± .116
Weight (kg)	6	67.00 ± 13.1
Dinamo_Max_Ext (kg/cm^2^)	6	39.66 ± 13.23
Dinamo_Max_Flex (kg/cm^2^)	6	38.00 ± 11.91
ULFI (score)	6	1.41 ± 2.20
QuickDASH (score)	6	7.19 ± 12.88
QuickDash_Work (score)	6	4.16 ± 7.56
QuickDash_Sport (score)	6	10.41 ± 16.61

Dinamo: dinamometry values; Max: maximun; Ext: extensión; Flex: flexion; ULFI: Upper Limb Functional Functional Index; QuickDash: QuickDash assement value.

Tables 2 and 3 show the descriptive analysis of the indirect variables obtained by each device. Table 2, the values registered by the sEMG, shows the mean of the maximum values normalised by muscle and movement sub-phase. Table 3 shows the values obtained by the accelerometer, based on the variation of each module vector by sub-phases.

**Table 2:** **Descriptive analysis of the maximum sEMG normlised values (%) by muscle and phase**

	T1	T2	T3	T4	T5
	Mean ± SD	Mean ± SD	Mean ± SD	Mean ± SD	Mean ± SD
MaxHypo (%)	4.5 ± 4.2	45.06 ± 20.5	35.6 ± 17.6	12.4 ± 6.0	12.0 ± 6.8
MaxTen (%)	4.6 ± 6.6	48.5 ± 22.1	34.3 ± 18.6	15.0 ± 7.5	7.2 ± 8.4
Max1ºd (%)	2.2 ± 2.3	14.1 ± 7.2	12.9 ± 6.6	12.5 ± 7.5	4.6 ± 4.2
MaxFlexWrist (%)	2.2 ± 1.3	10.0 ± 6.6	11.0 ± 5.9	8.1 ± 8.7	3.9 ± 1.9
MaxFlexCarpi (%)	10.0 ± 11.9	26.7 ± 19.0	33.0 ± 15.0	26.0 ± 32.4	12.7 ± 15.3
MaxExtWirst (%)	3.1 ± 2.3	13.8 ± 6.8	12.2 ± 3.5	9.7 ± 3.2	7.6 ± 4.5

Max: maximun; Hypo: hypothenar muscles; Ten: Thenar Muscles; 1ºD: first dorsal interosseous muscle; FlexWrist: flexors of the wrist; FlexCarpi: flexor carpi ulnaris muscle; ExtWrist: extensors of the wrist; T1-T5: phase of movement.

**Table 3:** **Descriptive analysis of the variation values by module vector (g) and phase of ACC**

	T1	T2	T3	T4	T5
	Mean ± SD	Mean ± SD	Mean ± SD	Mean ± SD	Mean ± SD
Thumb (g)	14.87 ± 16.23	117.64 ± 19.45	17.27 ± 6.58	105.05 ± 21.92	13.62 ± 12.43
Index (g)	22.69 ± 30.57	86.29 ± 10.48	8.67 ± 2.10	90.32 ± 16.92	11.03 ± 9.45
Middle (g)	21.33 ± 32.40	80.92 ± 19.79	6.37 ± 2.19	85.25 ± 14.31	10.27 ± 10.12
Ring (g)	26.08 ± 37.78	92.12 ± 16.58	5.85 ± .96	88.83 ± 11.63	12.15 ± 11.00
Pinky (g)	16.38 ± 24.30	95.28 ± 19.89	9.57 ± 2.62	96.34 ± 12.85	10.37 ± 9.20
Palm (g)	6.82 ± 4.34	50.13 ± 5.36	6.44 ± 1.24	44.78 ± 15.80	9.00 ± 6.44

Thumb: thumb vector resultant; Index: index vector resultant; Middle: middle vector resultant; Ring: ring vector resultant; Pinky: pinky vector resultant; Palm: palm vector resultant; T1-T5: phase of movement.

Figure 4 shows two agreement patterns generated with the used devices. The first of these, the upper section in the figure, shows the pattern of acceleration variations on the basis of the maximum, mean and minimum values obtained by the module of the acceleration vector palm. Moreover, the lower section of the figure shows the normalised values obtained by the sEMG based on the flexor muscles of the wrist.

## Discussion

The reading equipment used for surface electromyography and accelerometry has proved a valuable tool for parameterising the functionality of the hand. This system complements the current evaluation scales and provides new variables of interest and study.

Using both devices simultaneously allows the identification of those variables which have greater relevance in the description of the movement. The use of synchronised devices during spherical grip brings new variables, such as the maximum values of ACC and sEMG, or their variation and other derivation of them. For example this variables provides information on the moments in which muscle activity is at a maximum and its relation to the time in which the accelerations occur. This allows us to perform a rehabilitation of greater accuracy since it can be used to identify the muscle with greater activation at each moment, giving way to a recovery phase.

The protocol used can, thanks to its specificity for the evaluation of the hand, be used to complement the different validated assessments of the hand. This fact allows the acceleration and electromyography variables to be recorded during the different functional evaluations, the main outcome variable of which is time.

The inclusion of data processing in the R software has allowed Acceleglove resampling, which has a lower frequency of sampling. The processing of data based on Matlab for the detection of the static phases was appropriate, since it allowed fragmentation of the movement in the periods indicated.

Similarly, the use of these peripherals, after analysing the results obtained from each reading, allows us to detect those elements which have changed. These may be tremors registered by the patterns of acceleration or very low values of muscular activity registered by the sEMG. This is achieved thanks to the precision offered and the level of reliability.

Tables 2 and 3 of the descriptive analysis of movement from electromyography and accelerometry shows great variability, focused primarily on muscle activity. This is due to the muscle activity of each subject and the effect of crosstalk (Mogk and Keir 2003). Muscle activity varies depending on the muscular development of each subject, according to the development of fibres. This variability is exacerbated because the sample size is small and it has not been possible to establish homogeneous values of these variables. In the case of accelerometer data, variability is due to the execution speed and the hand position adaptation for each movement and phase.

Hand movements have been analysed while performing different grips by registering the angular position of the 15 joints of the hand (Santello et al. 1998) using a glove fitted with CyberGlove sensors (Overview | cyberglovesystems.com 2012). This study suggests that control of hand posture is regulated by the synergies involved in the shape of the hand (principal components), making adjustments for the object being gripped.

There are many different contact areas for handgrips, totalling sixteen elements (Liu and Zhan 2012). All of these areas are involved in spherical gripping, correlating to maintain pressure on the object. The fingers exercise parallel pressure on the X and Y spatial axes, generating an envelope around the surface of the object. The contact areas are studied not only in relation to the hand, but also with regards to the shape, size and weight of different grips (Fu and Santello 2011). Moreover, for the purpose of spherical gripping (Cobos Guzmán et al. 2010), the degrees of freedom of movement are 1 to 3; these are provided by the wrist, the other element involved in holding the object.

Electromyographic analysis of the grips has been made comparatively between sEMG and needle electromyography (nEMG) in relation to force, this being related to electrical activity with the force produced (Kamavuako et al. 2009). The conclusion is that both types of electromyography correlate similarly with grips of force (such as spherical). In other words, the selection of a single muscle with nEMG provides valuable, proportionate data in relation to the force exerted on a given object.

Therefore, the main importance of the present study focuses in the combined use of both procedures, sEMG and ACC data, which have barely been used in the hand simultaneously in the analysis of the various tasks.

The duration of each reading was different, with a mean duration of 8.29 ± 0.35 seconds. This variation is due to the reaction speed of each subject and the execution speed. Both factors affect the duration of each movement phase. These elements do not influence the acceleration or electromyographic values;, variation peaks were used.

The use of surface electromyography in the hand is difficult to implement due to the large number of muscles involved and the limitations of the electrode surface. However, it can give an overall idea of the muscle groups involved in making hand movements, namely the index, thumb and pinky, since these are the regions for which sEMG is available.

The use of inertial sensors above the sEMG electrodes can generate an increase in signal noise due to electrical conduction and the elements of the system. However, the combined use of these devices provides additional information on hand movement, based on the muscle activity produced during movement of the different segments.

The small sample size prevented us from obtaining a larger number of correlations and establishing standardised values. This circumstance has allowed a thorough analysis of the data provided by each of the devices and the treatment of the signals.

The implementation of the protocol developed for other functional hand gestures is possible, as is the comparison of different exertions and the detection of a director vector as a single representative element of hand movement. This system could provide important information for the analysis and early detection of pathologies such as Parkinson’s, and could be used as a complementary element for evaluations in the hand.

## Conclusion

Parameterisation of the spherical grip was performed in real time based on positive electromyography and accelerometry values. We identified the module vector of the palm as the director vector in spherical gripping and the guide vector in movement, allowing fragmentation based on the results in five phases. The remaining data for variables for different phases were extracted based on the palm vector fragmentation, thus obtaining the value of the variables in the same time periods. All of this can be extrapolated given the similarity in position when manipulating a mouse.

## Abbreviations

IS: Inertial sensors
EMG: Electromyography
sEMG: Surface electromyography
nEMG: Neddle electromyography
MVC: Maximum voluntary contraction
ULFI: Upper limb functional index
ACC: Acceleglove.
